# OR13-002 Recessive mutations in CECR1, encoding adenosine deaminase 2 (ADA2), cause systemic and cutaneous polyarteritis nodosa (PAN)

**DOI:** 10.1186/1546-0096-11-S1-A264

**Published:** 2013-11-08

**Authors:** E Levy-Lahad, P Elkan-Navon, R Segel, SB Pierce, T Walsh, J Barash, S Padeh, A Zlotogorski, YY Berkun, JJ Press, M Mukamel, PJ Hashkes, LL Harel, M Tekin, F Yalcinkaya, O Kasapcopur, EF Emirogullari, MK Lee, RE Klevit, PF Renbaum, A Weinberg-Shukron, S Zeligson, D Marek-Yagel, M Shohat, A Singer, E Pras, AA Rubinow, Y Anikster, M-C King

**Affiliations:** 1 Shaare Zedek Medical Center, Jerusalem, Israel; 2 University of Washington, Seattle, USA; 3 Kaplan Medical Center, Rehovoth, Israel; 4 Sheba Medical Center, Tel Hashomer, Israel; 5 Hadassah Medical Center, Jerusalem, Israel; 6 Schneider Children’s Medical Center, Petach Tikvah, Israel; 7 Ankara University, Ankara, Turkey; 8 University of Miami, Miami, USA; 9 Istanbul University, Istanbul, Turkey; 10 Rabin Medical Center, Beilinson Hospital, Petach Tikvah, Israel; 11 Barzilai Medical Center, Ashkelon, Israel

## Introduction

Polyarteritis nodosa (PAN) is a systemic necrotizing vasculitis of middle-sized arteries, found in both adults and children. Disease pathogenesis is poorly understood. We identified multiple cases of systemic PAN and cutaneous PAN in families and individuals of Georgian-Jewish ancestry, consistent with autosomal recessive inheritance. While most cases (17/20) had childhood onset, cutaneous PAN could also initiate in middle age.

## Objectives

To determine the genetic basis of monogenic PAN.

## Methods

Exome sequencing of 4 affected individuals from 2 families was followed by targeted sequencing of 16 additional Georgian-Jewish cases and 6 Turkish pediatric cases of PAN. Mutations were assayed by protein structure analysis, expression in mammalian cells, biophysical analysis of purified protein, and enzymatic activity in patient sera.

## Results

Missense mutation CECR1 p.G47R (c.139G>A), in the gene encoding ADA2, was the only damaging variant homozygous in all 4 exomes. Of the 20 Georgian-Jewish patients, 19 were homozygous for this mutation and one was compound heterozygous for G47R and H391Y. One Turkish patient was compound heterozygous for G47V and W264S. In the Georgian-Jewish population, the frequency of G47R was 0.05, reflecting the high prevalence of PAN in this endogamous community. The other mutations were absent from ethnically matched controls.

ADA2 activity was significantly reduced in patient sera. Expression of mutant proteins in HEK293T cells yielded significantly reduced levels of secreted ADA2 and biophysical assays indicated reduced protein stability.

## Conclusion

We report mutations in the gene encoding ADA2 as the first genetic cause of a systemic vasculitis. ADA2 is the major extra-cellular ADA, so blood vessels may be particularly vulnerable to loss of its catalytic and immune growth factor activity.

## Disclosure of interest

None declared

